# Various tests of left neglect are associated with distinct territories of hypoperfusion in acute stroke

**DOI:** 10.1093/braincomms/fcac064

**Published:** 2022-03-17

**Authors:** Colin Stein, Lisa Bunker, Brian Chu, Richard Leigh, Andreia Faria, Argye E. Hillis

**Affiliations:** 1 Department of Neurology, School of Medicine, Johns Hopkins University, Baltimore, MD, USA; 2 Department of Radiology, School of Medicine, Johns Hopkins University, Baltimore, MD, USA; 3 Department of Physical Medicine & Rehabilitation, School of Medicine, Johns Hopkins University, Baltimore, MD, USA; 4 Department of Cognitive Science, Johns Hopkins University, Baltimore, MD, USA

**Keywords:** acute ischaemic stroke, hemispatial neglect, MRI, vascular territories

## Abstract

Hemispatial neglect is among the most disabling consequences of right hemisphere stroke. However, there is no consensus on the optimal assessments to identify neglect. We hypothesized that different tests for neglect given the same day (i) detect distinct aspects and types of neglect, (ii) are sensitive to different cognitive functions (beyond spatially specific processing) and (iii) are associated with distinct regions of hypoperfusion. We examined data from 135 participants with acute, right-hemispheric ischaemic stroke who received an MRI and neglect testing within 48 h of acute infarct in a cross-sectional study. The volume of infarct was calculated on diffusion-weighted imaging. We also scored severity and location of fluid-attenuated inversion recovery hyperintense vessels in six areas (anterior cerebral artery territory, posterior cerebral artery territory and four within the middle cerebral artery territory) to estimate the volume and location of hypoperfusion in acute stroke. Neglect tests included gap detection, scene copy, line bisection, line cancellation, oral reading and picture description. We found strong correlations between tests that evaluated viewer-centred processing, as well as strong correlations between tests that evaluated stimulus-centred processing. The error rate on different tests was associated with hypoperfusion in different vascular territories, even after controlling for the volume of an infarct. Our results confirm that it is essential to administer a battery of different tests of hemispatial neglect to capture various deficits in attention and spatially specific processing that underlies neglect. Our results also show the potential usefulness of hyperintense vessel ratings as an indication of dysfunction beyond the infarct, as the ratings (and not infarct volume) were highly associated with many clinical deficits. Finally, results underscore that diverse types of neglect are clinically important in acute stroke, as they reflect different areas of hypoperfused tissue, which may be salvageable in the absence of infarct in those areas. As such, neglect batteries may be useful for detecting patients with cortical hypoperfusion who are candidates for reperfusion therapies.

## Teaser

Hillis *et al*. tested 135 participants within 48 h of onset on a battery of tests of hemispatial neglect. They found that different tests for neglect, given the same day, detect distinct aspects and types of neglect and are associated with distinct regions of hypoperfusion, independently of the volume of an infarct.

## Graphical Abstract

**Graphical Abstract:**
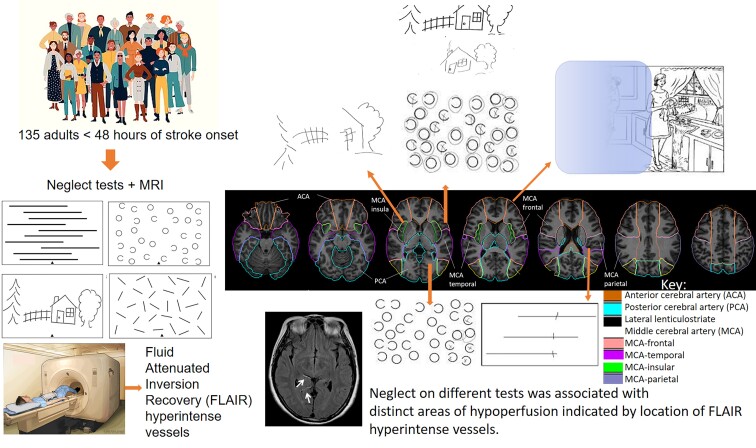

## Introduction

Many tests have been described to evaluate for hemispatial neglect after stroke. These tests are administered most frequently to patients after right hemisphere stroke because left neglect after right hemisphere stroke is more apparent than right neglect after left hemisphere stroke (but see Edmonds *et al.* regarding the frequency of each).^1^ Several investigators have noted that using more than one test of neglect is more sensitive than using only one,^2–4^ and some authors have reported that some tests are more sensitive than others.^5^ One plausible reason that a battery of different tests is more sensitive to neglect than a single test is that the tests evaluate different aspects of neglect or different types of neglect, so that only a battery could identify all the aspects or types. Indeed, a variety of distinct forms of neglect have been described, such as neglect specific to a single modality (e.g. visual, tactile, motor),^6^ personal versus extrapersonal,^7^ attentional versus intentional,^8^ altitudinal versus horizontal^9,10^ or neglect specific to a particular reference frame (e.g. object-centred, stimulus-centred and viewer-centred;^11,12^ or egocentric versus allocentric^13,14^). It seems likely that different assessments or different scoring would be required to identify each type of neglect. Furthermore, no test reflects the degree of competence in only one cognitive function. In addition to spatially specific processing, performance on various tests also reflects (to various degrees) sustained attention/vigilance, executive function, reading (e.g. written word recognition and sometimes the cognitive processes underlying oral reading), constructional skills, picture/figure/letter recognition, visually guided reaching and so on. In fact, Corbetta and Shulman^15^ have proposed that hemispatial neglect requires two simultaneous deficits: impaired spatially specific processing (due to deficits in either hemisphere) and impaired vigilance (more common after right hemisphere damage). This proposal might account for the greater frequency/severity of neglect after right hemisphere stroke.

However, another possible reason that multiple tests are more sensitive than a single test of hemispatial neglect is that neglect fluctuates, so that multiple assessments (whether the same or different tests) will be more likely to detect the impairment when it is present or more severe. Indeed, we have shown that changes in performance on tests of neglect in acute stroke reflect changes in blood flow to specific brain regions^16–18^. Others have reported that test–retest reliability of some tests of neglect is low even in chronic stroke.^19^

We hypothesized that different tests for neglect given on the same day, in the same testing session (<48 h of stroke onset) (i) detect distinct aspects and types of neglect and (ii) are sensitive to different cognitive functions (beyond spatially specific processing). It follows from the hypothesis that if various tests are differentially sensitive to non-spatial cognitive functions, they will also reflect dysfunction of distinct areas of the brain that are critical to these functions, at least acutely, before reorganization or recovery. In acute stroke, clinical deficits reflect areas of the dysfunctional brain, including areas that are markedly hypoperfused, as well as those that are infarcted.^20–22^ In acute stroke, volume, severity and location of hypoperfusion can be measured on MRI bolus-tracking perfusion or CT perfusion imaging. However, not all patients can have these images, often because of renal dysfunction or lack of intravenous access that hinder contrast administration. Hypoperfusion can also be evaluated without contrast with arterial spin labelling MRI. However, this modality is sensitive to motion artefact and other challenges and so is not widely available. However, MRI fluid attenuation inversion recovery (FLAIR) is often a standard sequence for stroke imaging and provides an alternative approach to estimating the location and volume of hypoperfusion, as hyperintense vessels on FLAIR usually indicate areas of low blood flow.^23–25^

We tested these hypotheses in a retrospective analysis of prospectively collected data. These data were collected as a part of a larger study of cognitive deficits in acute stroke and recovery. Because more neglect tests were consistently administered to participants with right hemisphere stroke than to those with left hemisphere stroke, we evaluated these hypotheses in participants with right hemisphere stroke, within 48 h of testing. We included all 135 patients who met the criteria (a convenience sample).

## Materials and methods

### Participants

We examined data from 135 participants with acute, right-hemispheric ischaemic stroke who received an MRI and neglect testing within 48 h of acute infarct at the Johns Hopkins Hospital or Johns Hopkins Bayview Medical Center. This cohort was prospectively enrolled into a parent study from 2013 to 2019. We attempted to test all right hemisphere stroke patients within 48 h of stroke onset who consented to the study, irrespective of whether or not neglect was observed. Patients received an MRI scan and testing after the administration of any thrombolysis or other intervention. Patients who either did not receive a FLAIR scan or received an unreadable FLAIR scan were excluded from the study. Patients with bilateral lesions, history of dementia or other neurological disease involving the brain, uncorrected premorbid hearing or visual loss, or impaired level of consciousness or ongoing sedation, were excluded. We did not exclude participants with visual field cuts on examination because neglect can mimic homonymous hemianopia, as demonstrated by visual field testing indicating left homonymous hemianopia in the presence of normal visual evoked responses in the left visual field.^26^ The mean age was 59.7 years (SD = 13.3; range = 24–88). Of the 135, 66 (48.9%) were female. The study was approved by the Johns Hopkins Institutional Review Board. All participants provided informed consent for the study, in accord with the Declaration of Helsinki.

### MRI protocol and analyses

All participants had axial diffusion weighted imaging (DWI) and FLAIR scans on 1.5 or 3 T MRI as part of clinical care. Trained technicians manually traced lesions on DWI scans using MRIcron or MRIcroGL (available at nitric.org); tracings on each slice were verified by experienced researchers. SPM12 (Statistical Parameter Mapping; https://www.fil.ion.ucl.ac.uk/spm/software/spm12/) routines were used to warp each patient’s DWI b0 image to a healthy geriatric adult template to create a lesion map. The volume of infarct was calculated on the normalized lesion map (in mm^3^) using NiiStat (https://www.nitrc.org/projects/niistat/).

**Figure 1:**
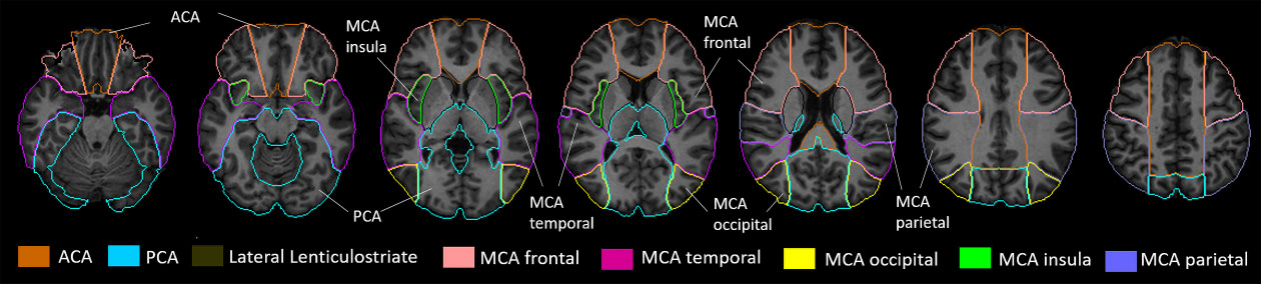
**Digital three-dimensional brain MRI arterial territories atlas.** This atlas is derived from Liu *et al*. (https://www.biorxiv.org/content/10.1101/2021.05.03.442478v2) Subdivisions of biological importance in the MCA territory were based on probabilistic maps and/or anatomical landmarks. ACA and PCA territories were not separated, as they were less commonly affected territories.

Two trained study team members scored severity and location of FLAIR hyperintense vessels (FHVs) using methods described by Reyes *et al*.^25^ On each slice of the FLAIR sequence, FHVs were scored on a 3-point scale (i.e. 0–2) in six vascular regions—anterior cerebral artery (ACA) territory, posterior cerebral artery (PCA) territory and the middle cerebral artery (MCA) territory divided into frontal (MCA frontal), temporal (MCA temporal), insular (MCA insular) and parietal regions (MCA parietal). These areas correspond to vascular territories defined in a recently developed arterial territory atlas (https://www.biorxiv.org/content/10.1101/2021.05.03.442478v2) (Fig. 1), using subdivisions of biological importance in the MCA territory, based on probabilistic maps and/or anatomical landmarks. ACA and PCA territories were not separated into subregions, as they were less commonly affected territories. A vascular region was assigned a score of 0 if there were no hyperintense vessels in that area on any slice and a score of 1 if there were one to two hyperintense vessels on one or two slices. The area was given a score of 2 if there were three or more FHV on one slice or if there were FHV on three or more slices. Scores for each area were totalled, for up to 12 possible points for the affected hemisphere. We then multiplied the total FHV score by 16 to estimate the volume of hypoperfusion, based on an earlier study showing that FHV rating ×16 estimates the volume of hypoperfusion.^25^ Following training on scoring FHV, two raters scored a sample of 32 FLAIR scans for the presence of FHV in each of the six vascular regions. Point-to-point agreement for the scores for each region, as well as the summed totals, was 93.1%.

### Neglect measures

Visuospatial neglect was measured using the following tests: gap detection, scene copy, line bisection, line cancellation, oral reading and picture description. Not all participants completed all of the tasks, due to time constraints in the acute setting. Each task, besides line bisection/cancellation and picture description, was scored in two separate ways to capture viewer-centred versus stimulus-centred neglect (SCN), described in the following paragraphs.

The gap detection task^27^ (*N* = 129) required participants to circle all complete circles and cross out (with an X) all of the circles with gaps. There were 30 circles in total, with 15 circles on each side of the page. Ten circles had a gap on the left side, and 10 had a gap on the right side. The remaining stimuli were full circles. Two types of errors were recorded: (i) stimuli to the left of any marked stimulus that was not marked at all (left viewer-centred neglect; VCN) or (ii) stimuli with a left gap that was circled, i.e. marked incorrectly as a full circle (left SCN).

**Figure 2:**
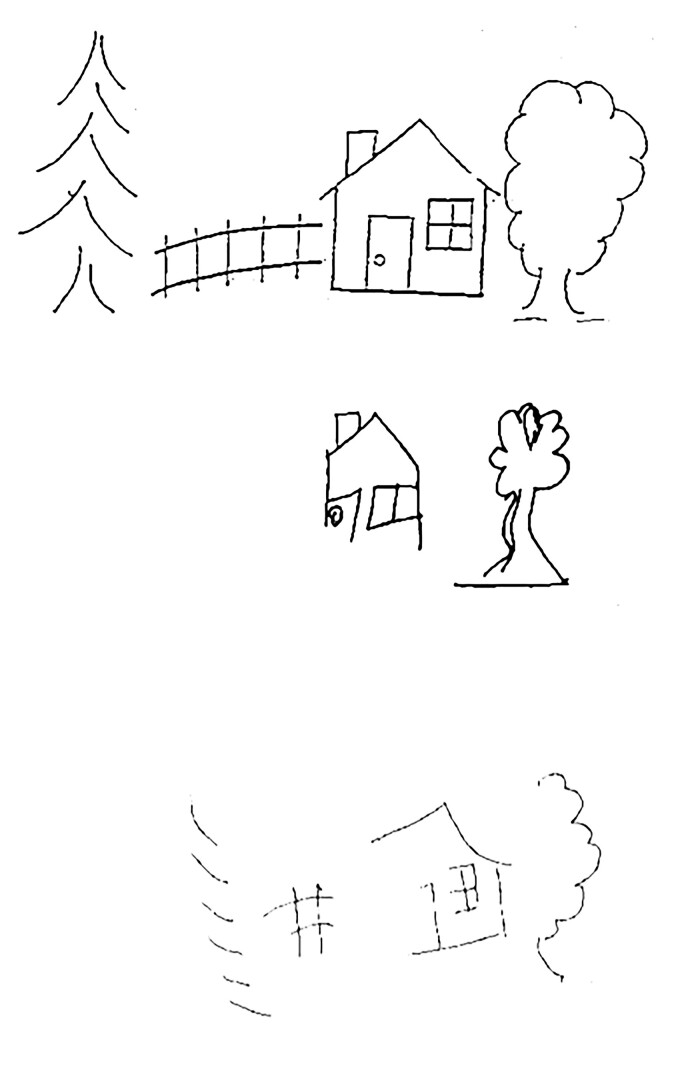
**Distinct patterns of performance in copying a scene**. *Top*: Scene that was shown to participants to copy. *Middle*: Viewer-centred neglect, characterized by omitting figures in the left side of the view. *Bottom*: Stimulus-centred neglect, characterized by omitting the left half of the scene, irrespective of the side of the view.

Copying of the ‘Ogden scene’^28^ (*N* = 132), required participants to copy a scene that consisted of a coniferous tree, a fence, a house and a deciduous tree (shown from left to right side of the page). The picture had 36 total components (pen strokes), with 16 components on the left side and 20 on the right side of the page. Within the house and the two trees, 14 components were on the left and 14 components on the right side of the stimuli. Each omitted component was scored as an error; and misplaced or distorted components were scored as half an error. Errors across all 36 components were used to indicate VCN (Fig. 2, top), whereas errors on the house and two trees were used to indicate SCN (Fig. 2, bottom).

Line cancellation^29^ (*N* = 66) required participants to cross out every line, of 28, on a landscape page, 8.5 × 11 inches presented: (i) at the midsagittal plane, (ii) 45° to the right, (iii) 45° to the left of the viewer’s midsagittal plane. The percentage of lines omitted on the left-most 21 lines of each page was scored. We selected the left-most 21 of 28 lines because no participant omitted the right-most 7 lines on any page (but one omitted 21 lines to the left of the 7 right-most lines).

Line bisection (*N* = 124) required drawing a line through the middle of a 10-inch horizontal line, thereby bisecting it in half. The error was measured as per cent of the line that was presumably neglected (deviated to the right).

Oral reading of words and sentences (*N* = 51) required participants to orally read 30 single words split over two columns—one on the left side of the page (15 items) and one on the right side of the page (15 items)—as well as five single-line sentences (flush left) of varying lengths (i.e. 6–8 words spanning in a single line across the page). All of the words can be made into another word by changing or omitting the first or last letters (e.g. darn could be read as ‘barn’ or ‘dark’ by erring on the first or last letter; rant could be read as ‘ant’ or ‘ran’ by omitting the first or last letter). To score VCN, we calculated the percentage of words in the left column and left sides of the sentence that were omitted. To score SCN, we calculated the percentage of words (alone or in sentences) that had errors on the left side but preserved one or more letters at the end (right side) (e.g. rant read as ‘ant’ or ‘pant’ or even ‘mint’ or ‘sit’).

Picture description (*N* = 8) required participants to describe everything happening in the ‘cookie theft’ picture from the Boston Diagnostic Aphasia Examination,^30^ which is now part of the National Institutes of Health Stroke Scale.^31^ Although not part of the scoring of either of the parent tests, we have reported that this description can be a sensitive test to both right neglect after left hemisphere stroke and left neglect after right hemisphere stroke.^32^ The description is scored for the number of content units (CUs) (concepts frequently expressed by healthy controls in describing this picture).^33^ There are 30 possible CUs on the left side of the paper and 23 possible CUs on the right side of the paper. Some CUs (e.g. disaster, kitchen) are not scored as right or left CUs. The ratio of left:right CUs is significantly higher in the left hemisphere stroke patients compared with controls (and right hemisphere stroke) and significantly lower in right hemisphere stroke patients compared with controls (and left hemisphere stroke patients). Controls, on average, have a ratio close to one.^32^

### VCN versus SCN

As noted previously, a subset of the tests that were administered is scored in such a way as to distinguish VCN versus SCN errors.

VCN was defined as (i) significantly (*P* < 0.05 here and elsewhere) more errors of omission on the left, compared with the right side of the page/viewer in line cancellation, gap detection, copying a scene or sentence reading.

SCN was defined as (i) significantly more errors in detecting the left gaps in circles (of 10) than right gaps in circles (of 10) in the gap detection tests; (ii) significantly more errors on the left, compared with the right, sides of stimuli, irrespective of the side of the page, in copying a scene or (iii) significantly more errors in reading the initial letters of words than final letters of words (on either side of the page), in reading sentences (e.g. *house* read as ‘mouse’ or ‘use’ versus ‘hound’ or ‘how’).

Right-biased errors on other tasks, such as line bisection, can reflect either VCN or SCN, unless the single stimulus is presented and scored on the left versus the right side of the view. Errors in omitting left-sided items in a picture description, which has several items (e.g. children, cookie jar), likely reflect VCN, but it would be difficult to detect errors in processing just the left side of each of these items versus the entire item altogether. There were no tasks administered to specifically evaluate tactile, motor, personal versus extrapersonal, attentional versus intentional or object-centred neglect. SCN (as we use it in this paper) could reflect SCN or object-centred neglect. Object-centred neglect (as we use it) refers to neglect of the left side of the canonical representation of the word, such as the initial letters in reading aloud normal print, vertical words, mirror-reversed words, spelling aloud and so on.^34^

### Statistical analysis

All analyses were carried out in STATA (version 16.1). Pearson correlations were used to identify correlations between scores on the various neglect tests. We applied Bonferroni corrections for multiple comparisons. We also identified correlations between scores on each test and volume of infarct and volume of hypoperfusion, after Bonferroni correction. To determine the independent contributions of FHV ratings in each area to the neglect score on each test, we carried out multivariable linear regression. In each model, the dependent variable was the score on each test, and the independent variables were the FHV in each of the six regions. Because the volume of infarct and/or total volume of hypoperfusion were correlated only with scores on copying and line bisection, we included these variables in the models where copying or line bisection score was the dependent variable. Statistical tests were two-tailed, and the alpha-level that was used to determine significance was *P* < 0.05 (after Bonferroni correction).

### Data availability

Anonymized data used for these analyses are available upon request from the senior author, by email, argye@jhmi.edu.

## Results

### Correlations between tests

After correcting for multiple comparisons, viewer-centred scoring of the gap detection test was significantly correlated with line cancellation (*r* = 0.78; *P* < 0.0001), line bisection (*r* = 0.37; *P* < 0.00001), *viewer-centred scoring* of copying a scene (*r* = 0.50; *P* < 0.0001) and *viewer-centred scoring* of oral sentence reading (*r* = 0.64; *P* < 0.0001), but not with *stimulus-centred scoring* of gap detection, copying a scene or oral reading (*r* = 0.23–0.33; ns). Similarly, viewer-centred scoring of copying a scene correlated with viewer-centred scoring of oral reading (*r* = 0.57; *P* < 0.0001) and line cancellation (*r* = 0.84; *P* < 0.0001), and line bisection (*r* = 0.33, *P* = 0.0003). *Stimulus-centred scoring* of the gap detection test significantly correlated with *stimulus-centred scoring* of copying of a scene (0.48; *P* < 0.0001) only. These results provide converging evidence that VCN and SCN are dissociable, so that tests sensitive to each are highly correlated with one another. Deviation in line bisection correlated only with scores on viewer-centred assessments.

Only eight participants had transcribed picture descriptions, allowing us to compute a ratio of left:right CUs. However, this left:right ratio strongly negatively correlated with viewer-centred scoring of copying a scene (*r* = −0.85; *P* = 0.0077), gap detection (stimulus-centred scoring, *r* = −0.87; *P* = 0.0048) and line bisection (*r* = −0.92; *P* = 0.0096). That is, more errors on copying or gap detection or greater deviation to the right in line bisection was associated with a smaller left:right content ratio (i.e. fewer CUs on the left than right mentioned in describing the picture).

### Correlations between test scores and volume of infarct/hypoperfusion and age

In univariate analyses, only scores on one test, line bisection, correlated with total infarct volume (*r* = 0.29; *P* = 0.0029). Scores on only one test, copying a scene (with viewer-centred scoring) correlated with a total volume of hypoperfusion estimated with FHV ratings (*r* = 0.54; *P* = 0.0055). Therefore, we included volume of infarct in the linear regression model for line bisection, and total volume of hypoperfusion in the model for copying a scene, viewer-centred errors. Age did not correlate significantly with any of the scores and was, therefore, not included in the multivariable models.

### The contribution of distinct regions of hypoperfusion to scores on each test

There were no FHV in the left hemisphere in any of the participants, so FHV ratings all refer to those in the right hemisphere. Multivariable linear regression, with scores on each test as the dependent variable and FHV rating in each area as the independent variables, revealed that hypoperfusion in different areas influenced scores on various tests of neglect (see Table 1 for coefficients and confidence intervals). Only the FHV rating in the right MCA temporal region significantly influenced the viewer-centred score on the gap detection test (*t* = 2.36, *P* = 0.020), such that higher FHV ratings were associated with more viewer-centred errors. In contrast, only the FHV rating in the right MCA frontal region significantly influenced both (i) the stimulus-centred errors on gap detection (*t* = 2.53; *P* = 0.013) and (ii) left-right CU ratio in picture description (*t* = −3.70, *P* = 0.034), with higher scores associated with lower left-right CU ratios. Only the FHV rating in MCA-insular regions significantly influenced copying a scene, stimulus-centred errors (*t* = 2.15; *P* = 0.034). Only FHV in PCA territory significantly influenced line cancellation errors (*t* = 3.67; *P* = 0.001), and only FHC in MCA-parietal regions significantly influenced the degree of deviation on line bisection (*t* = 2.57; *P* = 0.011). None of the FHV ratings, independently or together, influenced the number of viewer-centred or stimulus-centred errors on oral sentence reading.

**Table 1:** Areas of hypoperfusion significantly associated with scores on each task

Task	Area of FHV rating significantly associated	Coefficient	SE	95% confidence interval
Left:right content unit ratio	MCA frontal	−0.46	0.13	(−0.86, −0.065)
Viewer-centred errors in gap detection	MCA temporal	0.038	0.016	(0.0061, 0.070)
stimulus-centred errors in gap detection	MCA frontal	0.059	0.023	(0.013, 0.11)
Stimulus-centred errors in copying a scene	MCA insular	0.051	0.024	(0.0040, 0.098)
Line cancellation	PCA	0.091	0.025	(0.041, 0.14)

For test scores that correlated with the volume of infarct or hypoperfusion, the multivariable regression models are shown in Tables 2 and 3. Although neither the volume of infarct alone nor FHV ratings in any of the regions were independently related to viewer-centred errors in copying a scene (perhaps because of insufficient power), the scores together explained much of the variance in viewer-centred copying errors [*F*(7,17) = 3.07; *P* = 0.028; *r*2 = 0.56; Table 2]. In contrast, deviation in line bisection was independently related to both the total volume of hypoperfusion and FHV rating in the right MCA parietal region (Table 3). Together, the independent variables accounted for a significant amount of variation in rightward deviation in line bisection [*F*(7,95) = 2.86, *P* = 0.0095; *r*^2^ = 0.17].

**Table 2:** Variables that together (but not independently) influenced viewer-centred errors (omissions) in copying a scene [*F*(7,17) = 3.07; *P* = 0.028; *r*2 = 0.56]

	Coef.	SE	*T*	*P*-value	(95% CI)
HP volume	−0.000037	0.00063	−0.06	0.95	(−0.0014, .0013)
HP ACA	2.28	11.18	0.20	0.84	(−21.32, 25.87)
HP PCA	4.58	31.94	0.14	0.89	(−62.80, 71.96)
HP MCA frontal	15.67	11.93	1.31	0.21	(−9.49, 40.84)
HP MCA temporal	−3.44	9.87	−0.35	0.73	(−24.26, 17.38)
HP MCA parietal	8.26	10.96	0.75	0.46	(−14.87, 31.38)
HP MCA insular	1.65	11.75	0.14	0.89	(−23.13, 26.43)
Constant	3.44	2.65	1.30	0.21	(−2.15, 9.04)

Coef., coefficient; SE, standard error; HP, hypoperfusion; ACA, anterior cerebral artery territory; PCA, posterior cerebral artery territory; MCA, middle cerebral artery territory.

**Table 3:** Variables that influenced deviation on line bisection

% Deviation	Coef.	SE	*T*	*P*-value	(95% CI)
**Infarct volume**	**0.000091**	**0.000037**	**2.42**	**0.017**	**(0.0000162, 0.000165)**
HP in ACA	1.80	1.89	0.96	0.34	(−1.94, 5.55)
HP in PCA	−0.085	2.60	−0.03	0.97	(−5.25, 5.08)
HP in MCA frontal	−1.37	1.79	−0.77	0.45	(−4.93, 2.18)
HP in MCA temporal	−1.28	1.26	−1.02	0.31	(−3.78, 1.22)
**HP in MCA parietal**	**4.11**	**1.49**	**2.76**	**0.007**	**(1.15, 7.07)**
HP in MCA insula	1.67	1.87	0.89	0.37	(−2.05, 5.38)

Bolded results are statistically significant. Coef., coefficient; SE, standard error; HP, hypoperfusion; ACA, anterior cerebral artery territory; PCA, posterior cerebral artery territory; MCA, middle cerebral artery territory.

The number of participants who contributed a left:right CU score in picture description (*n* = 8) was inadequate to conduct linear regression. However, in the univariate analysis even with this small number, there was a significant correlation between this ratio and FHV in the MCA-frontal area only (*r* = −0.87; *P* = 0.0048) after correcting for multiple comparisons.

## Discussion

We found that scores on tests that were designed to evaluate for VCN correlated with each other and with line bisection, while scores on tests designed to evaluate for SCN correlated with one another. Line bisection could be either a viewer-centred task or a stimulus-centred task because the middle of the line is the centre with respect to the view and the stimulus (assuming the page is presented directly in front of the participant, as it was in this study). However, viewer-centred processing is often considered a dorsal visual stream function,^35^required for computing ‘where’ something is with respect to the viewer and ‘how to’, for example, reach for it or act upon it. Since line bisection required the individual to move one’s pen to the stimulus and mark the centre of the line, it likely relied on dorsal stream processing. In contrast, a task of judging whether a line is accurately bisected or not might rely on stimulus-centred processing, which is proposed to be a function of the ventral visual stream, critical for knowing what something is, irrespective of its location with respect to the viewer. Alternatively, line bisection can be considered an ‘intentional’ task, whereas judging the accuracy of line bisection might be considered an ‘attentional’ task.^8,36^ Intentional neglect (impaired movement toward or reaching toward a contralesional stimulus) versus attentional neglect (perception of the contralesional side) have been shown to dissociate, sometimes with very clever paradigms.^8,37^ For example, a task in which the patient has to move a mouse upside down on the bottom of a table to mark a target on the screen so that reaching to the right moves the cursor to the left, which can distinguish between the two. Some patients have left intentional neglect, failing to move the mouse to the left with a leftward arm movement, whereas others have left attentional neglect, failing to move the mouse to the right towards a stimulus on the left of the screen. We did not include tasks that would distinguish these types of neglect. We also did not administer tests to distinguish visuospatial, representational^38^ and personal neglect,^5^ or those to evaluate neglect in other modalities (tactile, motor, auditory) or other dimensions (altitudinal versus horizontal), although these types can also dissociate as described earlier.

Interestingly, only the score on line bisection was associated with the total volume of infarct; and infarct volume accounted for only about 8% of the variance in line deviation (*r*^2^ = 0.08). The volume of infarct was not independently associated with line deviation after controlling for areas of FHV. Similarly, only scores on copying a scene (with viewer-centred scoring) correlated with the total volume of hypoperfusion estimated with FHV ratings, and the volume of hypoperfusion accounted for only about 29% of the variance on this score (*r*^2^ = 0.29). Although the volume of hypoperfusion explained more of the variance in neglect than the volume of infarct on most tests (see also Olsen *et al.*^20^), clearly other factors such as the location of hypoperfusion influenced scores. We found that different tests (even within the set of viewer-centred or stimulus-centred subgroups) were associated with distinct areas where there were FHVs, indicating that impaired scores were likely associated with different areas of hypoperfusion. Although these results indicate differences in areas of hypoperfusion, we cannot be certain of the precise location of hypoperfusion indicated by the FHVs. That is, we do not know, for example, if FHV in the MCA-parietal area indicates that the hypoperfusion is in the parietal cortex. We are currently carrying out a study on a large, independent sample of patients who had both dynamic contrast perfusion-weighted imaging and FLAIR (but no neglect testing) to determine if the area of hyperintense vessels corresponds to a specific area of hypoperfusion. Irrespective of the specificity of the location, it is clear that distinct areas of dysfunction in the right hemisphere influence performance on different tests of neglect. These results are consistent with our hypothesis that different tests for neglect given at the same time not only detect distinct aspects and types of neglect but also reflect hypoperfusion of distinct areas of the brain.

In this study, age did not correlate with any of the scores on neglect tests. However, previous studies have shown that age influences severity of neglect,^39^ likely because age is associated with atrophy^40^ and white matter hyperintensities,^41^which both affect neglect severity. However, we may have had inadequate power or inadequate variance in age to detect the effect of age.

Limitations of this study include the fact that not all patients were administered all of our tests of neglect. Time for assessment is often limited during the first 2 days of stroke, so we were not always able to administer all of the tests. We could not include patients with a reduced level of consciousness or inability to provide informed consent in the first 48 h after stroke (usually those with the largest strokes). In addition, we added the picture description as part of the battery much later than the other tests, so that only a small number of participants had that test in addition to the others. In addition, we did not test for every type of neglect, such as representational or personal neglect^5^ or intentional or motor–exploratory neglect,^36,42^ and did not administer tests of motor neglect,^43,44^ tactile neglect,^6,45^ behavioural assessments,^46^ computer assessments^47^ or virtual reality assessments,^48^ all of which have some strengths. Instead, we wished to show that even different ‘paper and pencil’ tasks evaluate different aspects of neglect and rely on different brain regions. Another limitation is that we were only able to estimate the volume and site of hypoperfusion, as described in previous studies.^23–25^ As mentioned earlier, we are currently evaluating how precisely the FHV rating reflects the location of hypoperfusion. Future studies will localize the area of hypoperfusion associated with scores on each test using dynamic contrast perfusion-weighted MRI and/or CT perfusion (another contrast-based map of hypoperfusion) and set thresholds to identify dysfunctional tissue (e.g. 6 s delay in time-to-peak arrival of contrast).

This study focused on hypoperfusion rather than infarct. This focus was based on our previous studies showing that in acute stroke the severity and type of neglect are more associated with the areas of cortical hypoperfusion than with the area of infarct and that resolution of cortical hypoperfusion results in resolution of neglect.^16–18,49,50^ However, we found that in every case of cortical infarct, the FHV (when present) was in the same vascular distribution subregion as the infarct. For example, all 11 participants with right MCA temporal FHV also had infarcts in the right MCA temporal area, and no participants without right MCA temporal infarcts had FHV in the right MCA temporal area (*χ*^2^ = 96.0); *P* < 0.0001 (see Supplementary material for table of all associations between the area of infarct and area of FHV). However, there were cases of FHV in areas without infarct in the same area. All but one of these cases were participants with subcortical infarcts with cortical FHV. Of the 63 participants with lenticulostriate infarcts, 46 (73%) had FHV in one or more cortical regions. There was a significant association between subcortical infarct and the presence of cortical FHV (*χ*^2^ = 5.7; *P* = 0.017).

Despite its limitations, this study demonstrates that it is essential to administer a battery of different tests of hemispatial neglect to capture distinct deficits in spatially specific processing. Although a particularly small number of patients had transcribed picture descriptions, the high correlations between left and right CUs in describing the cookie theft picture and more traditional tests of neglect (copying, gap detection, line bisection) indicate that it is a useful rapid bedside test of neglect. Nearly every acute stroke patient is asked to describe the picture as part of the NIH Stroke Scale, and the number of left and right CUs can be checked off as the person speaks (so that it does not need to be transcribed for clinical use).^29^ Our results also confirm the potential usefulness of the FHV rating as an indication of dysfunction beyond the infarct, as the ratings (and not infarct volume) were highly associated with many clinical deficits. Finally, results underscore that these distinct types of neglect are clinically important in acute stroke because they reflect different areas of hypoperfused tissue, which may be salvageable in the absence of infarct in those areas. Thus, neglect batteries may be useful for detecting patients with cortical hypoperfusion who are candidates for reperfusion therapies.

## Supplementary Material

fcac064_Supplementary_Data

## Abbreviations

ACA =: anterior cerebral artery
FHVs =: FLAIR hyperintense vessels
FLAIR =: fluid attenuation inversion recovery
MCA =: middle cerebral artery
PCA =: posterior cerebral artery
SCN =: stimulus-centred neglect
VCN =: viewer-centred neglect
