# Supplementary material for: Various tests of left neglect are associated with distinct territories of hypoperfusion in acute stroke
Source: Brain Commun. 2022 Mar 17;4(2):fcac064. doi: 10.1093/braincomms/fcac064 (PMC8977645; doi:10.1093/braincomms/fcac064)
Supplement: fcac064_Supplementary_Data [file fcac064_supplementary_data.docx]

Supplemental Table 1. Associations Between Area of Infarct and Area of FLAIR Hyperintense Vessels

Area Chi Square p-value

Anterior Cerebral Artery Territory 1.34 p=0.25

Posterior Cerebral Artery Territory No patient had FHV in PCA territory

Middle Cerebral Artery (MCA)

MCA Frontal 1.94 p=0.16

MCA Temporal 96.0 p<0.0001

MCA Parietal 2.81 p=0.09

MCA Insular 2.03 p=0.15
